# Implementation of an Antibiotic Stewardship Initiative in a Large Urgent Care Network

**DOI:** 10.1001/jamanetworkopen.2023.13011

**Published:** 2023-05-11

**Authors:** Edward Stenehjem, Anthony Wallin, Park Willis, Naresh Kumar, Allan M. Seibert, Whitney R. Buckel, Valoree Stanfield, Kimberly D. Brunisholz, Nora Fino, Matthew H. Samore, Rajendu Srivastava, Lauri A. Hicks, Adam L. Hersh

**Affiliations:** 1 Division of Infectious Diseases and Epidemiology, Intermountain Health, Salt Lake City, Utah; 2 Intermountain Urgent Care, Intermountain Health, Salt Lake City, Utah; 3 Office of Research, Intermountain Health, Salt Lake City, Utah; 4 System Pharmacy Services, Intermountain Health, Salt Lake City, Utah; 5 Intermountain Health Delivery Institute, Intermountain Health, Salt Lake City, Utah; 6 Department of Internal Medicine, Division of Epidemiology, University of Utah School of Medicine, Salt Lake City; 7 Department of Pediatrics, Division of Pediatric Inpatient Medicine, University of Utah School of Medicine, Salt Lake City; 8 Office of Antibiotic Stewardship, Division of Healthcare Quality Promotion, Centers for Disease Control and Prevention, Atlanta, Georgia; 9 Department of Pediatrics, Division of Infectious Diseases, University of Utah School of Medicine, Salt Lake City

## Abstract

**Importance:**

Urgent Care (UC) encounters result in more inappropriate antibiotic prescriptions than other outpatient setting. Few stewardship interventions have focused on UC.

**Objective:**

To evaluate the effectiveness of an antibiotic stewardship initiative to reduce antibiotic prescribing for respiratory conditions in a UC network.

**Design, Setting, and Participants:**

This quality improvement study conducted in a UC network with 38 UC clinics and 1 telemedicine clinic included 493 724 total UC encounters. The study compared the antibiotic prescribing rates of all UC clinicians who encountered respiratory conditions for a 12-month baseline period (July 1, 2018, through June 30, 2019) with an intervention period (July 1, 2019, through June 30, 2020). A sustainability period (July 1, 2020, through June 30, 2021) was added post hoc.

**Interventions:**

Stewardship interventions included (1) education for clinicians and patients, (2) electronic health record (EHR) tools, (3) a transparent clinician benchmarking dashboard, and (4) media. Occurring independently but concurrent with the interventions, a stewardship measure was introduced by UC leadership into the quality measures, including a financial incentive.

**Main Outcomes and Measures:**

The primary outcome was the percentage of UC encounters with an antibiotic prescription for a respiratory condition. Secondary outcomes included antibiotic prescribing when antibiotics were not indicated (tier 3 encounters) and first-line antibiotics for acute otitis media, sinusitis, and pharyngitis. Interrupted time series with binomial generalized estimating equations were used to compare periods.

**Results:**

The baseline period included 207 047 UC encounters for respiratory conditions (56.8% female; mean [SD] age, 30.0 [21.4] years; 92.0% White race); the intervention period included 183 893 UC encounters (56.4% female; mean [SD] age, 30.7 [20.8] years; 91.2% White race). Antibiotic prescribing for respiratory conditions decreased from 47.8% (baseline) to 33.3% (intervention). During the initial intervention month, a 22% reduction in antibiotic prescribing occurred (odds ratio [OR], 0.78; 95% CI, 0.71-0.86). Antibiotic prescriptions decreased by 5% monthly during the intervention (OR, 0.95; 95% CI, 0.94-0.96). Antibiotic prescribing for tier 3 encounters decreased by 47% (OR, 0.53; 95% CI, 0.44-63), and first-line antibiotic prescriptions increased by 18% (OR, 1.18; 95% CI, 1.09-1.29) during the initial intervention month. Antibiotic prescriptions for tier 3 encounters decreased by an additional 4% each month (OR, 0.96; 95% CI, 0.94-0.98), whereas first-line antibiotic prescriptions did not change (OR, 1.00; 95% CI, 0.99-1.01). Antibiotic prescribing for respiratory conditions remained stable in the sustainability period.

**Conclusions and relevance:**

The findings of this quality improvement study indicated that a UC antibiotic stewardship initiative was associated with decreased antibiotic prescribing for respiratory conditions. This study provides a model for UC antibiotic stewardship.

## Key Points

**Question:**

What is the association between a multifaceted antibiotic stewardship initiative with antibiotic prescribing for respiratory conditions in a large urgent care (UC) network?

**Findings:**

This quality improvement study of 493 724 total UC encounters found a decrease in antibiotic prescribing for respiratory conditions from 48% (baseline) to 33% (intervention), with reductions of 22% at the start of the intervention and 5% per month throughout the 1-year intervention period.

**Meaning:**

This study’s findings suggest that a multifaceted antibiotic stewardship initiative was associated with reduced antibiotic prescribing for UC respiratory conditions, and that such initiatives in large UC networks may decrease inappropriate antibiotic prescribing.

## Teaser

This quality improvement study compares antibiotic prescribing rates for respiratory conditions before, during, and after implementation of a multifaceted antibiotic stewardship initiative in a large urgent care network.

## Introduction

The majority of antibiotic prescriptions in the United States result from outpatient encounters; up to 30% may be unnecessary.^1,2^ Despite awareness of problems associated with antibiotic overuse (eg, antibiotic resistance and antibiotic-associated adverse effects),^3^ there has been only modest improvement in the rate of unnecessary prescribing over time.^2^

Urgent care (UC) is one of the fastest growing sites of outpatient care delivery in the United States, with the encounter volumes increasing by 50% or more in recent years.^4,5^ These UC encounters result in more antibiotic prescriptions overall and more unnecessary antibiotic prescriptions compared with other outpatient settings.^6^ Infectious conditions, especially respiratory tract infections, which often lead to inappropriate antibiotic prescribing, are the most common types of diagnoses managed in UC.^7^

Recognition that unnecessary antibiotic prescribing is common in outpatient settings led the Centers for Disease Control and Prevention (CDC) to develop the Core Elements of Outpatient Antibiotic Stewardship.^8^ This guidance provides a framework for outpatient stewardship implementation. Most outpatient stewardship interventions have been designed and evaluated in primary care (PC) settings or emergency departments (EDs), with few interventions being applied specifically to UC.^9,10,11^ Urgent care incorporates features common to PC settings (eg, uncomplicated low-acuity respiratory conditions) and EDs (eg, expanded hours, walk-in visits, rotating clinicians, and expectations for fast turnaround). Therefore, stewardship programs that best meet the needs of patients and clinicians in UC settings should understand and incorporate these characteristics.

Substantial variability in rates of antibiotic prescribing for respiratory conditions exists between clinics and clinicians across the Intermountain Healthcare (IH) UC system.^7^ To improve prescribing, we developed an antibiotic stewardship program specifically designed for UC settings based on CDC Core Elements. The objective of the present study was to evaluate the association of this program with antibiotic use for respiratory conditions in UC encounters.

## Methods

### Setting and Study Design

Intermountain Health is a nonprofit, integrated, health care delivery system that includes 24 hospitals and more than 185 outpatient clinics throughout the Mountain West. During the quality improvement study, IH operated 38 UC clinics, including 32 for patients of all ages (InstaCare) and 6 providing care exclusively to children younger than 18 years (KidsCare). InstaCare and KidsCare clinics are predominantly staffed by physicians. In addition, IH operates a direct-to-consumer telemedicine UC clinic (Connect Care) primarily staffed by advanced practice clinicians (APCs). This study followed the Standards for Quality Improvement Reporting Excellence (SQUIRE) reporting guideline. The IH institutional review board approved this study and waived the need for obtaining informed consent for this minimal risk, evidence based, quality improvement study. The study is registered at ClinicalTrials.gov.^12^

A pre-post quality improvement study design was used to evaluate the association of a systemwide antibiotic stewardship initiative with antibiotic prescribing for respiratory conditions in the IH UC network. The prespecified primary and secondary analyses compared a 12-month baseline period (July 1, 2018, through June 30, 2019) with a 12-month intervention period (July 1, 2019, through June 30, 2020). In addition, we included a 12-month sustainability period (July 1, 2020, through June 30, 2021) to evaluate subsequent prescribing trends.

### Intervention

To inform the intervention, the study team (eAppendix in Supplement 1) performed site visits to assess the UC environment and to replicate the patient experience. Patient and clinician interviews were performed to identify barriers and possible solutions to improve antibiotic prescribing.^13^ Our interventions were developed using 4 categories based on the CDC Core Elements: education focused on UC clinicians and patients (eAppendix in Supplement 1); electronic health record (EHR; Cerner) tools to assist clinicians in ordering antibiotic prescriptions correctly and more efficiently documenting encounters; a transparent clinician benchmarking dashboard; and media targeting patients and clinicians. The Box provides a full description of the interventions.

Box. **Components of Antibiotic Stewardship Intervention Deployed in the IH UC Network and Concomitant Independent Antibiotic Utilization Financial Incentive**

**Intervention Domain: Education**

**Specific Intervention for Clinician**

UC antibiotic stewardship champion (P.W.) performed in-clinic education (peer-to-peer) and was a resource for clinicians
Handbook included all guidelines, patient educational material, and metric methods
Monthly update lectures at regional UC meetings and UC email newsletter
Updated clinical guidelines
Internal podcasts with antibiotic stewardship leaders
Clinician facing antibiotic stewardship webpage, including all project resources
Systemwide antibiotic stewardship screensavers

**Specific Intervention for Patient**

Symptomatic therapies checklist
Watchful waiting or delayed antibiotic prescribing document
Patient facing antibiotic stewardship webpage
Folding antibiotic education brochure placed in clinic lobby and examine rooms

**Intervention Domain: EHR Tools**

**Specific Intervention for Clinician**

Azithromycin prescribing justification alert
Addition of delayed antibiotic prescriptions in the EHR
Creation of templated notes for common respiratory conditions to assist in documentation, billing, and ordering appropriate medications
Updated prepopulated antibiotic orders with guideline concordant regimens

**Intervention Domain: Clinician Antibiotic Prescribing Dashboard**

**Specific Intervention for Clinician**

Transparent antibiotic prescribing dashboard that included all clinicians, clinics, and system level antibiotic prescribing metrics allowing for peer comparison
Dashboard engagement was facilitated by including a dashboard link in all emails, within the EHR, and biannual data review with UC leaders

**Intervention Domain: Media**

**Specific Intervention for Patient and Clinician**

Local or regional television and radio interviews
Print media: newspaper articles, IH circulars
Facebook and Twitter posts
Commitment posters and waiting room antibiotic stewardship messaging via table tents, desk wraps, and floor stickers

**Intervention Domain: Financial Incentive^a^**

**Specific Intervention for Clinician**

UC clinicians given the goal to individually prescribe antibiotics in fewer than 50% of their respiratory encounters. This target was identified by a preintervention analysis that showed 50% to be the median respiratory antibiotic prescribing rate for the UC service line.^7^

Abbreviations: EHR, electronic health record; IH, Intermountain Health; UC, urgent care.

^a^
Not a formal component of the intervention.

### Financial Incentive

The IH UC service line has annual quality measures tied to compensation. If quality measures are met by clinicians, they are eligible to receive additional compensation. Occurring in parallel and independent of the development of the antibiotic stewardship interventions, IH UC leadership introduced the antibiotic prescribing measure into their bundle of quality measures,^14^ with an individual target of lower than 50% prescribing (Box).

### Study Outcomes

Data were electronically extracted from the EHR for each encounter and included patient-reported demographic characteristics, clinician specialty (MD, DO, or APC), discharge *International Classification of Diseases, Tenth Revision, Clinical Modification* (*ICD-10-CM*) codes, and antibiotic prescriptions ordered. Each encounter was categorized into 1 of 5 clinical categories (respiratory, skin or skin structure, gastroenterology, genitourinary, and other) based on *ICD-10-CM* codes.^7^ The respiratory category was further subcategorized and assigned tiered groups based on whether antibiotics were indicated: tier 1, antibiotics indicated (eg, pneumonia); tier 2, antibiotics sometimes indicated (eg, sinusitis); and tier 3, antibiotics not indicated (eg, bronchitis).^1^ If diagnosis codes from multiple tiers were present, the category defaulted to the lowest tier (eg, when tier 2 and tier 3 codes were present, the category was defined as tier 2). Encounters were excluded if no *ICD-10-CM* code was billed, if codes from multiple clinical categories were present (eg, respiratory and genitourinary), or if the clinician totaled fewer than 25 visits during the study period.

Antibiotic prescriptions generated or administered (eg, intravenous antibiotics) during UC encounters were captured as electronic orders, and topical or inhaled formulations were excluded. Delayed antibiotic prescriptions were created in the EHR for antibiotics traditionally used for sinusitis and acute otitis media (AOM), allowing for direct measurement of their use. Delayed antibiotic prescriptions counted as an antibiotic prescription in all measures.

The primary study outcome was the change in antibiotic prescribing for respiratory conditions between the baseline and intervention periods. Secondary outcomes included the change in antibiotic prescribing for tier 3 respiratory conditions and the use of first-line antibiotics for AOM, sinusitis, and pharyngitis. First-line antibiotics were defined as penicillin or amoxicillin for pharyngitis and amoxicillin or amoxicillin-clavulanate for acute sinusitis and AOM. Additional outcomes included the percentage of respiratory conditions prescribed azithromycin and the use of delayed prescriptions for AOM and sinusitis.

Balancing measures related to the potential unintended consequences of the intervention included patient satisfaction and ED visits or hospitalizations within 14 days of a UC respiratory condition. Patient satisfaction was assessed using data from an IH patient satisfaction survey administered by Press Ganey.^15^ Data were extracted for the “Overall rating of care received during your visit” on a scale of 1 (very poor) to 5 (very good). Mean patient satisfaction scores for respiratory conditions were evaluated from January 1, 2019, through June 30, 2020, based on data availability.

### Statistical Analysis

An interrupted time series model was used to assess the association of the intervention with the primary and secondary outcomes.^16^ Binomial generalized estimating equations were used to account for clustering among the clinicians and in the clinic. For the prespecified primary and secondary outcomes comparing the intervention with baseline period, regression models included time-based terms to estimate the slope per month for antibiotic prescribing in the baseline period, the change in prescribing at the start of the intervention, and the change from the period before the intervention to the intervention period. The same approach was used to compare the sustainability period with the intervention period. For each model, an odds ratio (OR) was estimated for antibiotic prescribing per 1 month change in the baseline period (the baseline period slope), the change in odds of antibiotic prescribing from the baseline period to intervention (the immediate change associated with the intervention initiation during July 2019), and the OR for antibiotic prescribing per 1 month change during the intervention (the intervention period slope). This OR was calculated using the linear combination of the terms for baseline slope and the change in slope over time from baseline to the intervention using estimate statements in PROC GENMOD. Because the intervention period overlapped with the onset of the COVID-19 pandemic (March 2020), we performed a sensitivity analysis to exclude any association with study outcomes by limiting the intervention period to July 1, 2019, through February 28, 2020. All modeling was performed in SAS, version 9.4 (SAS Institute Inc). Statistical significance was defined as a 2-sided *P* < .05 or 95% CIs excluding 1.

## Results

The baseline period included 493 724 total UC encounters, of which 207 047 (41.9%) were respiratory conditions. The intervention period included 471 136 total UC encounters, of which 183 893 (39.0%) were respiratory conditions. Among respiratory conditions, patient demographic characteristics were similar between baseline and intervention periods, including age (mean [SD], 30.0 [21.4] vs 30.7 [20.8] years), female sex (56.8% vs 56.4%), and race (92.0% vs 91.2% White). The intervention period showed an increase in the proportion of Connect Care visits (3.8% at baseline to 8.4% during the intervention) with a corresponding decrease in InstaCare and KidsCare visits. There was no change in the proportion of visits by MD or DO clinicians compared with APCs. Clinical and demographic characteristics for the baseline and intervention periods are compared in Table 1.

**Table 1.** Characteristics of Antibiotic Prescribing and Respiratory Conditions Among Urgent Care Encounters During Baseline (July 1, 2018, to June 30, 2019), Intervention (July 1, 2019, to June 30, 2020), and Sustainability (July 1, 2020, to June 2021) Periods

Characteristic	No. (%)		
	Baseline	Intervention	Sustainability
All encounters	493 724	471 136	391 608
Encounters receiving antibiotic prescription	164 147 (33.3)	123 893 (26.3)	87 744 (22.4)
Respiratory conditions	207 047 (41.9)	183 726 (39.0)	95 221 (24.3)
Respiratory conditions receiving antibiotic prescription	98 867 (47.8)	61 243 (33.3)	24 277 (25.5)
Among respiratory encounters			
Female	117 602 (56.8)	103 626 (56.4)	55 083 (57.8)
Male	89 445 (43.2)	80 100 (43.6)	40 138 (42.2)
Patient age, mean (SD), y	30.0 (21.4)	30.7 (20.8)	32.3 (19.9)
Patient aged <18 y	67 504 (32.6)	53 403 (29.1)	21 222 (22.3)
Race			
American Indian or Alaska Native	1376 (0.7)	1248 (0.7)	742 (0.8)
Asian	3239 (1.6)	2989 (1.6)	1311 (1.4)
Black or African American	2456 (1.2)	2302 (1.3)	1376 (1.6)
Multiple	513 (0.3)	468 (0.3)	271 (0.3)
Native Hawaiian or Pacific Islander	2630 (1.3)	2496 (1.4)	1407 (1.5)
Other, declined, or not provided	6249 (3.0)	6660 (3.6)	4013 (4.2)
White	190 584 (92.0)	167 563 (91.2)	86 101 (90.4)
Ethnicity			
Hispanic	21 931 (10.8)	21 316 (11.8)	12 410 (13.3)
Not Hispanic	180 925 (87.4)	158 013 (86.0)	80 132 (84.2)
Other, declined, or not provided	4191 (2.0)	4397 (2.4)	2679 (2.8)
Clinic type			
Connect Care	7894 (3.8)	15 413 (8.4)	10 945 (11.5)
InstaCare	185 224 (89.5)	158 232 (86.1)	81 102 (85.2)
KidsCare	13 929 (6.7)	10 081 (5.5)	3174 (3.3)
Encounter with MD or DO	186 575 (90.1)	167 394 (91.1)	90 247 (94.8)
Tier 1 encounter	20 472 (9.9)	16 332 (8.9)	4974 (5.2)
Tier 1 encounter with antibiotic	19 851 (97.0)	15 578 (95.4)	4619 (92.9)
Tier 2 encounter	99 348 (48.0)	74 895 (40.8)	40 645 (42.7)
Tier 2 encounter with antibiotic	62 719 (63.1)	38 753 (51.7)	16 564 (40.8)
Tier 3 encounter	87 227 (42.1)	92 499 (50.3)	49 602 (52.1)
Tier 3 encounter with antibiotic	16 297 (18.7)	6912 (7.5)	3094 (6.2)
First-line antibiotic^a^	54 503 (70.7)	35 902 (74.5)	13 960 (72.5)
Delayed prescription^b^	NA	7930 (24.8)	3137 (24.6)
Azithromycin prescription	10 597 (5.1)	2176 (1.2)	799 (0.8)

Abbreviations: DO, doctor of osteopathic medicine; MD, medical doctor; NA, not applicable.

^a^ First-line antibiotic prescriptions among sinusitis, otitis media, and pharyngitis encounters when an antibiotic was prescribed. First-line antibiotics were defined as penicillin or amoxicillin for pharyngitis and amoxicillin or amoxicillin-clavulanate for acute sinusitis and acute otitis media. Antibiotic allergies were not taken into consideration.

^b^ Delayed prescription use among sinusitis and acute otitis media encounters when an antibiotic was prescribed. Delayed prescriptions were unable to be measured during the baseline period.

### Primary Outcome

Among respiratory conditions, antibiotic prescribing decreased from 47.8% during the baseline period to 33.3% in the intervention period (Table 1). Table 2 provides a summary of the interrupted time series analyses, and fitted models are shown in the Figure. Antibiotic prescribing did not change during the baseline period (OR, 0.99; 95% CI, 0.99-1.00; *P* = .11). During the initial month in which the intervention was implemented (July 2019), a 22% reduction in antibiotic prescribing (OR, 0.78; 95% CI, 0.71-0.86; *P* < .001) was observed. Antibiotic prescriptions continued to decrease by 5% each month during the course of the intervention (OR, 0.95; 95% CI, 0.94-0.96; *P* < .001). Similar decreases in antibiotic prescribing were observed across all clinic types (eg, InstaCare, KidsCare, and Connect Care). All clinics showed a decrease in antibiotic prescribing for respiratory conditions (range, −4.8% to −37.5%). Of clinicians who were present in both the baseline and intervention periods, 95% had a decrease in their individual antibiotic prescribing for respiratory conditions. During the baseline period, 38.5% of clinicians had a prescribing rate higher than 50%, compared with 10.2% during the intervention period (ie, only approximately 10% of clinicians did not meet the quality measure for the financial incentive). In a sensitivity analysis excluding the onset of the COVID-19 pandemic from the intervention period, the monthly decrease in antibiotic prescribing was slightly higher (OR, 0.93; 95% CI, 0.93-0.94 vs OR, 0.95; 95% CI, 0.94-0.96).

**Table 2.** Summary of ITS Analysis Evaluating the Association of the Intervention With Antibiotic Prescribing in the Intermountain Urgent Care Network

Population	Baseline period		Intervention rollout		Intervention period		Sustainability rollout		Sustainability period	
	OR for antibiotic prescribing/mo (95% CI)	*P* value	Change in OR for antibiotic prescribing from baseline to intervention (95% CI)	*P* value	OR for antibiotic prescribing/mo (95% CI)	*P* value	Change in OR forantibiotic prescribing from intervention to sustainability (95% CI)	*P* value	OR for antibiotic prescribing/mo (95% CI)	*P* value
All respiratory conditions	0.99 (0.99-1.00)	.11	0.78 (0.71-0.86)	<.001	0.95 (0.94-0.96)	<.001	0.90 (0.83-0.97)	.01	1.00 (1.00-1.01)	.24
Tier 3 respiratory conditions	0.99 (0.97-1.00)	.04	0.53 (0.44-0.63)	<.001	0.96 (0.94-0.98)	<.001	0.95 (0.81-1.11)	.50	1.01 (1.00-1.03)	.048
First-line prescriptions^a^	1.01 (1.00-1.02)	.10	1.18 (1.09-1.29)	<.001	1.00 (0.99-1.01)	.48	0.94 (0.80-1.05)	.30	1.00 (0.99-1.01)	.72

Abbreviations: ITS, interrupted time series; OR, odds ratio.

^a^ First-line antibiotic prescriptions among sinusitis, otitis media, and pharyngitis encounters where an antibiotic was prescribed. First-line antibiotics were defined as penicillin or amoxicillin for pharyngitis and amoxicillin or amoxicillin-clavulanate for acute sinusitis and acute otitis media. Antibiotic allergies were not taken into consideration.

**Figure.**
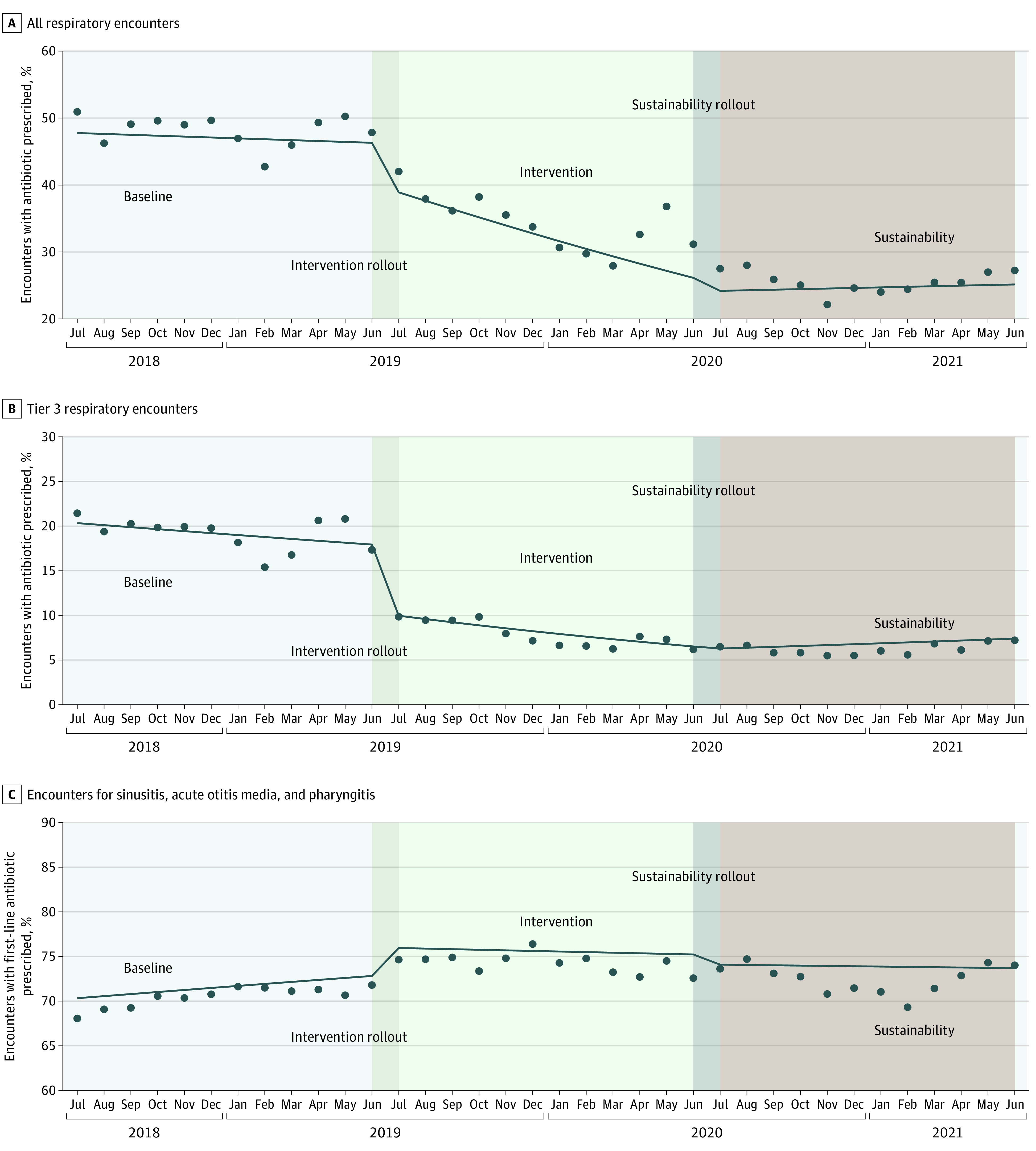
Fitted Interrupted Time Series Models for Baseline, Intervention, and Sustainability Periods The intervention was implemented July 1, 2019. Plots show the change in antibiotic prescribing rates for all respiratory encounters (A), all tier 3 respiratory encounters (B), and first-line antibiotics for sinusitis, acute otitis media, and pharyngitis encounters when an antibiotic was prescribed (C). The circles indicate the observed percentage of encounters receiving an antibiotic per month; dark blue lines indicate the fitted interrupted time series model.

### Secondary Outcomes

Among tier 3 respiratory encounters, antibiotic prescribing decreased from 18.7% during the baseline period to 7.5% in the intervention period (Table 1). Tier 3 antibiotic prescribing was already slightly decreasing during the baseline period (OR, 0.99; 95% CI, 0.97-1.00; *P* = .04); however, when the intervention was activated, a 47% reduction in antibiotic prescribing was observed for tier 3 encounters (OR, 0.53; 95% CI, 0.44-0.63; *P* < .001) in its initial month. Antibiotic prescriptions for tier 3 encounters continued to decrease by 4% each month during the course of the intervention (OR, 0.96; 95% CI, 0.94-0.98; *P* < .001) (Table 2; Figure).

First-line antibiotic prescriptions for AOM, sinusitis, and pharyngitis increased from 70.7% during the baseline period to 74.5% during the intervention period (Table 1). First-line antibiotic prescriptions were stable during the baseline period with an OR of 1.01 (95% CI, 1.00-1.02; *P* = .10). When the intervention was implemented, an 18% increase in first-line antibiotic prescribing was observed (OR, 1.18; 95% CI, 1.09-1.29; *P* < .001), but no further changes were observed over time (OR, 1.00; 95% CI, 0.99-1.01; *P* = .48) (Table 2; Figure). The percentage of respiratory conditions where azithromycin was prescribed decreased from 5.1% during the baseline period to 1.2% during the intervention period. Among sinusitis and AOM encounters, when an antibiotic was prescribed, a delayed prescription was used 24.8% of the time during the intervention period.

### Balancing Measures

Among respiratory conditions, patient satisfaction changed minimally. During the baseline period, the mean response rating was 4.4 (of 2533 surveys) compared with 4.3 (of 2189 surveys) during the intervention period. For patients who received an antibiotic, the baseline period mean response rating was 4.5 compared with 4.4 during the intervention. For patients who did not receive an antibiotic, the baseline period mean response rating was 4.2 compared with 4.2 during the intervention.

Hospitalizations within 14 days of a UC encounter occurred in 0.4% of encounters during the baseline period compared with 0.5% of encounters during the intervention period. This small increase was observed among tier 1 encounters (0.3% for baseline vs 0.4% for intervention), in which antibiotic prescribing did not change. Encounters in EDs within 14 days of a UC encounter (with no subsequent hospitalization) occurred in 8.1% of encounters during the baseline period and 8.6% of encounters during the intervention period. This small increase was observed after tier 1 UC encounters (6.5% for baseline vs 7.2% for intervention) and tier 2 encounters (7.2% for baseline vs 7.7% for intervention). There was no change among tier 3 encounters (9.6% for baseline vs 9.6% for intervention).

### Sustainability Period

The 1-year sustainability period included 391 608 UC encounters, of which 95 221 (24.3%) were respiratory conditions. Patient demographic characteristics were similar to the baseline and intervention periods. A greater proportion of patients visited Connect Care during the sustainability period (11.5%) compared with the intervention period (8.4%). Antibiotic prescribing for respiratory conditions was 25.5% in the sustainability period (vs 33.3% during intervention) (Table 1). Interrupted time series modeling showed a continued decrease in antibiotic prescribing at the start of the sustainability period, with minimal change thereafter (Table 2; Figure). Minimal change was observed with tier 3 prescribing (6.2% for sustainability vs 7.5% for intervention); first-line antibiotic therapy for AOM, sinusitis, and pharyngitis (72.5% for sustainability vs 74.5% for intervention); delayed prescriptions for AOM or sinusitis (24.6% for sustainability vs 24.8% for intervention), and azithromycin prescriptions (0.8% for sustainability vs 1.2% for intervention) (Table 1 and Table 2; Figure).

## Discussion

We developed an antibiotic stewardship program for UC in a large integrated health system. By the end of its initial 1-year implementation period, we observed an absolute reduction in antibiotic prescribing for respiratory conditions of 15%. Reductions in antibiotic prescribing were observed across all clinic sites and among nearly all clinicians. Improvements were also realized in antibiotic selection and prescribing for tier 3 respiratory conditions. Despite the substantial reduction in antibiotic prescribing, balance measures of ED visits and hospitalizations within 14 days of the UC encounter and patient satisfaction scores remained unchanged following the introduction of the intervention.

This study adds to growing evidence about effective outpatient stewardship programs, including those focused on UC settings.^9,10,11,17,18,19^ Features of successful programs include the use of audit and feedback, clinician and patient education, EHR tools, and peer comparison or benchmarking. The interventions used in our program were integrated into the UC service line in a large health system.^20^ Although the antibiotic stewardship interventions themselves were novel for this health system, within this context, clinicians were familiar with several of the core components of the program, including regular updates to clinical guidelines that adhere to evidence-based practice, clinical decision support tools embedded in the EHR, and regular performance evaluations using standardized practice metrics.

We found that antibiotic prescribing rates did not revert to baseline rates after the intervention period. Unlike inpatient stewardship programs, which generally require longitudinal budgeted financial support to sustain implementation, an ongoing challenge for outpatient stewardship is program durability after implementation.^18,21,22^ When outpatient stewardship interventions are incorporated into an integrated system already deeply engaged in quality initiatives, sustainability may be enhanced.

Although not an explicit feature of our intervention, UC leadership added achieving an antibiotic stewardship quality measure as part of clinician compensation, along with the other elements of our intervention. Financial incentives have been shown to have a modest positive effect on reducing antibiotic prescribing^23,24^ and may have contributed to our findings. Notably, financial incentives have not been featured in multiple previous studies of outpatient stewardship interventions, nor are they included in the CDC Core Elements.

The last 3 months of the intervention period overlapped with the onset of the COVID-19 pandemic. This overlap was associated with substantial health care disruptions and a reduction in outpatient antibiotic prescriptions nationwide.^25^ We and others have reported on the association of the pandemic with antibiotic prescribing during UC visits.^25,26,27^ Changes in the case mix of patients seeking care in UC centers could potentially have influenced prescribing rates. However, a sensitivity analysis conducted in the present study excluding the period of the pandemic showed no difference in the primary outcome.

Our study evaluated a novel HEDIS (Healthcare Effectiveness Data and Information Set) metric (namely, antibiotic use for respiratory conditions) for antimicrobial stewardship in the outpatient setting that avoids confounding due to changes in coding practices, and it is now endorsed by the National Committee for Quality Assurance.^14^ In addition to reductions in the overall rate of antibiotic prescribing for respiratory conditions, several other areas of antibiotic prescribing improved, including the use of azithromycin and delayed prescriptions. Because these areas were explicitly targeted by components of our intervention, they likely represent changes in clinical practice and were sustained beyond the intervention period.

We found no differences in several balancing measures of patient safety and patient satisfaction when comparing the baseline and intervention periods. This is important because of the relatively large reduction in antibiotic use for respiratory conditions that occurred with the intervention. Our observation that patient satisfaction scores were stable may provide reassurance to clinicians that practicing antibiotic stewardship is not associated with unintended consequences in patient satisfaction and other important dimensions of clinical care, including visit duration, both of which have been cited as barriers to mitigating antibiotic overuse.^28,29,30,31^ Assessing hospitalization rates after the implementation of an ambulatory antibiotic stewardship initiative is critical because small increases in hospitalizations could change the acceptability of these initiatives.

### Limitations

Our study has limitations. It was not a randomized design, which limits interpretation of causality. Our study was performed in 1 large integrated health system, a single geographic region, and required substantial resources. These factors limit its generalizability to other UC settings. The multifaceted nature of the intervention did not allow for determination of each component’s independent contribution to the measured outcomes. The findings regarding patient satisfaction were limited by the small sample size. Although our results suggest that there was no increase in severe disease as measured by ED visits and hospitalizations, because we did not prospectively enroll patients and assess symptoms over time, we cannot exclude the possibility that some illnesses not treated with antibiotics could have been prolonged. The intervention period was only 1 year; thus, its long-term sustainability and penetrance when confronted with changes in health care delivery demands are uncertain.

## Conclusion

The findings of this quality improvement study of a multifaceted UC antibiotic stewardship initiative in a large integrated health care system indicated a significant reduction in antibiotic prescribing for respiratory conditions by the end of the study period that was sustained with no detectable changes in unintended consequences. This study provides a model for UC stewardship in a large integrated health care system.

## Notes

Supplement 1. **eAppendix.** Study Team and Website Link

Supplement 2.
Data Sharing Statement
