# Supplementary material for: Implementation of an Antibiotic Stewardship Initiative in a Large Urgent Care Network
Source: JAMA Netw Open. 2023 May 11;6(5):e2313011. doi: 10.1001/jamanetworkopen.2023.13011 (PMC10176123; doi:10.1001/jamanetworkopen.2023.13011)
Supplement: Supplement 1. — eAppendix. Study Team and Website Link [file jamanetwopen-e2313011-s001.pdf]

Supplementary Online Content

Stenehjem E, Wallin A, Willis P, et al. Implementation of an antibiotic stewardship initiative in a large urgent care network. *JAMA Netw Open*. 2023;6(5):e2313011. doi:10.1001/jamanetworkopen.2023.13011

eAppendix. Study Team and Website Link

This supplementary material has been provided by the authors to give readers additional information about their work.

© 2023 Stenehjem E et al. *JAMA Network Open*.

eAppendix: Study Team and Website Link

Implementation of an Antibiotic Stewardship Initiative in a Large Urgent Care Network

Study Team:

Team Leaders:

1. Principle Investigator / Intermountain Health Antibiotic Stewardship Medical Director:

   Dr. Stenehjem was allocated 0.25 FTE for this project.

2. Principle Investigator / Pediatric Infectious Diseases and Antibiotic Stewardship Researcher, University of Utah:

   Dr. Hersh was allocated 0.25 FTE for this project.

   Drs. Stenehjem and Hersh were responsible for all components of the study, including content curation and implementation. Both investigators participated in monthly, regional urgent care service line meetings for approximately one year. The meetings were throughout Utah (drive time could range between 15 minutes and 4 hours) and average approximately 3 – 4 meetings per month. Some meetings were done remotely; most were done in person.

3. System Urgent Care Medical Director

   Dr. Wallin was not allocated any FTE specifically for this project, his involvement and participation fell under his medical director role.

   Dr. Wallin participated in all aspects of the study.

Other team members:

1. Implementation Scientist (0.1 FTE) – Our implementation scientist led our qualitative evaluation in preparation of our intervention. This qualitative work informed our intervention and consisted of interviews of patients, clinicians, and other urgent care staff members.
2. Urgent Care Stewardship Lead (0.2 FTE) – The urgent care stewardship lead was a practicing urgent care physician (family medicine) that was well respected in the clinic and had a very low antibiotic prescribing rate at baseline with excellent patient satisfaction scores. The stewardship lead participated in and led educational activities, coached clinicians with high prescribing rates, was a resource to clinicians, and was an active member of the team in all aspect of study design and implementation.
3. Project manager/coordinator (1.0 FTE) – The project manager coordinated and managed all aspects of the study including budget, IRB, timelines, meeting coordination, and educational tool development.

© 2023 Stenehjem E et al. *JAMA Network Open*.

4. Data analyst (0.3 FTE) – Intermountain employees a full-time data analyst specifically for antibiotic stewardship. Approximately 30% of the analyst's time was dedicated to the development of the urgent care antibiotic stewardship data platform.
5. Infectious diseases (ID) and antibiotic stewardship pharmacy manager (no dedicated FTE) – Our system ID pharmacy manager (and individual ID/stewardship pharmacists) were involved in reviewing guidelines (including antibiotic selection, dosing, and duration) and all modifications to the electronic health record antibiotic orders.
6. Intermountain Health employed medical informaticists (for electronic health record modifications), medical writers, marketing and communication specialists, and office of research staff were utilized in their employed roles and not provided additional funding for this work.

The supplement contains three ambulatory guidelines and two patient education documents that we feel the readers would find useful. All of these documents are available on our publicly facing website (https://intermountainhealthcare.org/health-information/health-library/antibiotic-stewardship/forphysicians/).

© 2023 Stenehjem E et al. *JAMA Network Open*.
