# Supplementary material for: Implementation of an Antibiotic Stewardship Initiative in a Large Urgent Care Network
Source: JAMA Netw Open. 2023 May 11;6(5):e2313011. doi: 10.1001/jamanetworkopen.2023.13011 (PMC10176123; doi:10.1001/jamanetworkopen.2023.13011)
Supplement: Supplement 2. — Data Sharing Statement [file jamanetwopen-e2313011-s002.pdf]

Data Sharing Statement

Stenehjem. Implementation of an Antibiotic Stewardship Initiative in a Large Urgent Care Network. *JAMA Netw Open*. Published May 11, 2023.
doi:10.1001/jamanetworkopen.2023.13011

Data
Data available: No
